# Prokaryote genome fluidity is dependent on effective population size

**DOI:** 10.1038/ismej.2017.36

**Published:** 2017-04-14

**Authors:** Nadia Andrea Andreani, Elze Hesse, Michiel Vos

**Affiliations:** 1 Department of Comparative Biomedicine and Food Science (BCA), University of Padova, Padua, Italy; 2 European Centre for Environment and Human Health, University of Exeter Medical School, University of Exeter, Penryn, UK; 3 Department of Biosciences, University of Exeter, Penryn, UK

## Abstract

Many prokaryote species are known to have fluid genomes, with different strains varying markedly in accessory gene content through the combined action of gene loss, gene gain via lateral transfer, as well as gene duplication. However, the evolutionary forces determining genome fluidity are not yet well understood. We here for the first time systematically analyse the degree to which this distinctive genomic feature differs between bacterial species. We find that genome fluidity is positively correlated with synonymous nucleotide diversity of the core genome, a measure of effective population size *N*_e_. No effects of genome size, phylogeny or homologous recombination rate on genome fluidity were found. Our findings are consistent with a scenario where accessory gene content turnover is for a large part dictated by neutral evolution.

## Results and discussion

Many bacterial species have been shown to exhibit extensive variation in gene repertoires, where a set of core genes shared by all strains are supplemented with a set of accessory genes that are only present in a subset of strains (Ochman *et al.*, 2000; Gogarten *et al.*, 2002; Tettelin *et al.*, 2005). Although accessory genome analyses are routinely performed in prokaryote genomics studies, whether certain genome characteristics are associated with particularly low or high genome fluidity has not been systematically tested. We here make use of the increasing availability of whole-genome sequences to, for the first time, perform a meta-analysis to (1) gauge the extent to which genome fluidity varies among different species and (2) test which genome characteristics best explain genome fluidity.

Methods to quantify pan-genome diversity are generally sensitive to the absence of rare accessory genes from genome samples. We therefore use the *φ* measure of genome fluidity that has been shown to be robust to sample size (Kislyuk *et al.*, 2011) (Supplementary Methods). This measure of genomic fluidity is defined as the ratio of unique gene families to the sum of gene families in pairs of genomes averaged over randomly chosen genome pairs from within a group of sampled genomes. Because it is vital to reliably score gene presence/absence and most available genomes are not sequenced to completion, we first verified that good quality (<150 contigs) non-closed genomes resulted in fluidity estimates comparable to those based on closed genomes (linear regression, *R*^2^=0.70, *P*<0.001; Supplementary Figure S1). Genome fluidity could be calculated for 90 free-living species for which five or more genomic data sets were available (3 archaea and 87 bacteria belonging to 15 major taxonomic groups, Supplementary Table S1). Only a single species was selected per genus to minimize phylogenetic bias. As estimates for individual species are dependent on genome selection and to a degree on the specifics of bioinformatics processing, they are not to be taken as absolutes and we will refrain from highlighting individual species, analysing broad patterns only.

**Figure 1:**
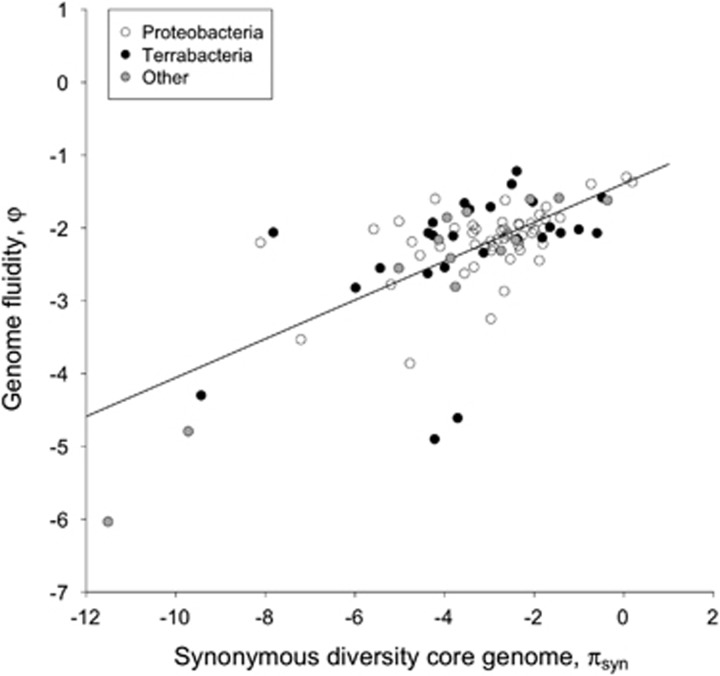
The genome fluidity statistic *φ* as a function of synonymous core genome nucleotide variation *π* for 90 free-living prokaryote species on a ln-ln scale. White dots: Proteobacteria, black dots: Terrabacteria (Actinobacteria, Firmicutes and Cyanobacteria), grey dots: other taxa.

Genome fluidity *φ* was plotted against synonymous nucleotide diversity of the core genome (*π*_syn_) on a natural log scale for all species (Figure 1), which showed a significant positive relationship (linear regression: ln(*φ*)=−1.39(0.12)+0.27(0.03) × ln(*π*); a: *t*=−11.61*** and b: *t*=8.59***, adjusted *R*^2^=0.45). No genetically monomorphic species with high gene content variation or species with diverse core genomes but limited variation in accessory gene content were found. The same analysis was performed for the genera *Pseudomonas* and *Streptococcus* for which multiple species genome sets are available (Supplementary Tables S2 and S3). All estimates of *φ* for these two genera were found to lie inside the 95% prediction interval of the relationship depicted in Figure 1 (Supplementary Figure S2), adding to the generality of our finding. A linear mixed-effects model was used with phylogenetic grouping included (group-dependent random intercepts) to test for the effect of genome size in addition to *π*_syn_ (fixed effects) (Table 1). This analysis was limited to the 77 species belonging to the broad Proteobacteria and Terrabacteria classifications. No effect of phylogeny or genome size (ranging from 0.9 to 10.2 Mb) on genome fluidity was found, but the positive relation with evolutionary divergence of the core genome remained highly significant (Table 1).

Interestingly, the intercept of the relationship of *φ* with *π*_syn_ is significantly different from zero (Table 1), indicating that accessory genomes diverge before single-nucleotide polymorphisms appear in the core genome. This finding supports the emerging view that changes in gene content occur at high rates relative to mutation in bacteria (Touchon *et al.*, 2009; Nowell *et al.*, 2014; Vos *et al.*, 2015; Wielgoss *et al.*, 2016). The uptake and loss of accessory genes is in part mediated via recombination of flanking homologous sequences (Polz *et al.*, 2013). To test whether the flexibility of the accessory genome is dependent on the rate of homologous recombination in the core genome, we compared *φ* estimates and *r*/*m* estimates (the probability that a nucleotide is changed as the result of recombination relative to point mutation) for 26 species that also featured in a meta-analysis of homologous recombination rate (Vos and Didelot, 2009). No significant relationship was detected (linear regression: *φ*=0.13(0.01)+0.01(0.01) × ln(*r*/*m*), a: *t*=9.78*** and b: *t*=0.54^NS^, adjusted *R*^2^=−0.03; Supplementary Table S4), confirming results of a previous analysis (Narra and Ochman, 2006).

The *φ* estimate only provides a general indication of genome fluidity as it ignores genome rearrangements or plasmids, and we cannot exclude the fact that elevated or decreased levels of genome fluidity are associated with some of the many phyla that could not be included in this analysis due to a lack of data. These caveats aside, the positive relationship of genome fluidity with synonymous diversity is highly significant. The synonymous nucleotide diversity equals two times the product of the mutation rate *μ* and effective population size *N*_e_ for haploid species. As variation in prokaryote mutation rate is believed to be relatively small (Lynch, 2010), *π*_syn_ can be taken as a proxy for *N*_e_. Large effective population size is expected to result in generally higher levels of genetic diversity due to neutral evolution (Kimura, 1984). The result of our cross-species meta-analysis is therefore consistent with the expectation that large *N*_e_ species exhibit greater accessory genome variation. A variety of studies have suggested that many gene content changes have only minor effects on fitness and are effectively neutral (Gogarten and Townsend, 2005; Baumdicker *et al.*, 2012; Haegeman and Weitz, 2012; Knöppel *et al.*, 2014), although it is clear that a proportion of gene gains and losses will be significantly deleterious or beneficial. To gain a full understanding of selection on the accessory genome, it will be vital to collect data on the distribution of fitness effects of gene content changes (Vos *et al.*, 2015).

## Figures and Tables

**Table 1:** Results of the linear mixed-effects model testing the additive effects of genome size and synonymous core genome diversity (*π*_syn_, ln-transformed) on accessory genome fluidity (*φ*, ln-transformed) with random intercepts fitted for each broad phylogenetic group (that is, Proteobacteria and Terrabacteria)

	*Parameter estimate±s.e.*^a^	F*-test*
Intercept	−1.64±0.18***, *t*=−8.87	
Genome size	−0.02±0.04^NS^, *t*=−0.42	*F*_1,4_=0.18, *P*=0.67
*π*_syn_	0.17±0.04***, *t*=4.05	*F*_1,4_=15.42, *P*<0.001
Phylogenetic group	<1% of total variance	

Abbreviation: NS, not significant.

a Note: significance of parameter estimates are based on Wald’s *t*-test, ***P<0.001.

The most parsimonious model was arrived at by sequentially deleting terms and comparing model fits using *F*-tests of likelihood ratios.
