# Supplementary material for: Prokaryote genome fluidity is dependent on effective population size
Source: ISME J. 2017 Apr 14;11(7):1719–21. doi: 10.1038/ismej.2017.36 (PMC5520154; doi:10.1038/ismej.2017.36)
Supplement: Supplementary Information [file ismej201736x1.docx]

**Supplementary Information (SI) “Prokaryote genome fluidity is dependent on effective population size”**

This document contains: Supplementary Methods (with Supplementary References), four Supplementary Tables and two Supplementary Figures.

**Supplementary Methods**

Genome multifasta files were manually downloaded from NCBI. Plasmid sequences were not included. We selected the highest quality genomes available for each species, including closed genomes, never using genomes consisting of more than 150 contigs. The freely available webserver odose (Vos et al 2013) (www.odose.nl) was used to perform analyses using both custom scripts and published software OrthoMCL (Li et al 2003) was used for a reciprocal best blast analysis to tabulate accessory gene distributions. Default settings were used for the e-value, 10^-5^, with a minimum protein length for inclusion in the analysis of 30 amino acids. Genes were aligned at the protein level using Muscle (Edgar 2004) implemented in TranslatorX (Abascal et al 2010) using the standard translation table. Alignments were trimmed using a custom python script. Orthologs of which the trimmed alignment was less than 90% in length of the untrimmed alignment were discarded. MEGA-CC (Kumar et al 2012) was used to calculate π_syn_. A MatLab script was used to calculate φ (http://ecotheory.biology.gatech.edu/downloads/genomic-fluidity-scripts). R (Team 2008) (version 3.2.3) was used for correlations, ANCOVA and generating graphs.

Table S1. Details of analysed species

| Species | Group | Phylum | Genome Size (Mb) | n genomes used | phi | var | pi syn |
| --- | --- | --- | --- | --- | --- | --- | --- |
| *Acetobacter pasteurianus* | Proteobacteria | Alphaproteobacteria | 3.34025 | 10 | 0.0211 | 2.7020E-04 | 0.00844 |
| *Achromobacter xylosoxidans* | Proteobacteria | Betaproteobacteria | 7.35915 | 8 | 0.1939 | 2.3000E-03 | 0.26969 |
| *Acinetobacter baumannii* | Proteobacteria | Gammaproteobacteria | 6.04643 | 25 | 0.1500 | 4.0607E-05 | 0.11018 |
| *Actinobacillus pleuropneumoniae* | Proteobacteria | Gammaproteobacteria | 2.27448 | 16 | 0.1053 | 1.6887E-05 | 0.05374 |
| *Aeromonas veronii* | Proteobacteria | Gammaproteobacteria | 4.73561 | 22 | 0.1093 | 2.2000E-03 | 0.18703 |
| *Aggregatibacter actinomycetemcomitans* | Proteobacteria | Gammaproteobacteria | 2.30907 | 13 | 0.1222 | 1.6459E-04 | 0.10336 |
| *Alteromonas mediterranea* | Proteobacteria | Gammaproteobacteria | 4.48094 | 9 | 0.1054 | 1.6694E-04 | 0.04597 |
| *Amycolatopsis mediterranei* | Terrabacteria group | Actinobacteria | 10.2367 | 6 | 0.0136 | 3.1210E-05 | 0.00008 |
| *Arcobacter butzleri* | Proteobacteria | delta/epsilon subdivisions | 2.34125 | 11 | 0.1268 | 5.1425E-05 | 0.07471 |
| *Bacillus anthracis* | Terrabacteria group | Firmicutes | 5.22866 | 25 | 0.1274 | 2.2000E-03 | 0.00040 |
| *Bacteroides fragilis* | FCB group | Bacteroidetes/Chlorobi group | 4.81151 | 25 | 0.2008 | 7.9192E-05 | 0.12358 |
| *Bifidobacterium longum* | Terrabacteria group | Actinobacteria | 2.26027 | 25 | 0.2475 | 2.8116E-05 | 0.08193 |
| *Bordetella bronchiseptica* | Proteobacteria | Betaproteobacteria | 5.26438 | 25 | 0.0726 | 1.4012E-04 | 0.02850 |
| *Brachyspira hyodysenteriae* | Spirochaetes | Spirochaetia | 3.03663 | 15 | 0.0604 | 1.6879E-05 | 0.02330 |
| *Burkholderia pseudomallei* | Proteobacteria | Betaproteobacteria | 7.24755 | 25 | 0.1121 | 9.1723E-05 | 0.00884 |
| *Campylobacter jejuni* | Proteobacteria | delta/epsilon subdivisions | 1.64148 | 25 | 0.1336 | 1.2610E-04 | 0.06937 |
| *Candidatus Pelagibacter ubique* | Proteobacteria | Alphaproteobacteria | 1.396 | 13 | 0.2548 | 1.3000E-03 | 1.21043 |
| *Cedecea neteri* | Proteobacteria | Gammaproteobacteria | 4.87644 | 6 | 0.2723 | 6.3000E-03 | 1.05842 |
| *Citrobacter freundii* | Proteobacteria | Gammaproteobacteria | 5.37133 | 8 | 0.2885 | 1.1500E-02 | 0.22886 |
| *Clostridium botulinum* | Terrabacteria group | Firmicutes | 3.00534 | 25 | 0.3596 | 3.6490E-04 | 0.68051 |
| *Comamonas testosteroni* | Proteobacteria | Betaproteobacteria | 5.37364 | 13 | 0.1624 | 1.8987E-04 | 0.15212 |
| *Corynebacterium diphtheriae* | Terrabacteria group | Actinobacteria | 2.48863 | 23 | 0.0965 | 1.6996E-05 | 0.04375 |
| *Coxiella burnetii* | Proteobacteria | Gammaproteobacteria | 2.03267 | 10 | 0.1333 | 3.9304E-04 | 0.00376 |
| *Dehalococcoides mccartyi* | Terrabacteria group | Chloroflexi | 1.38231 | 13 | 0.1328 | 1.6404E-04 | 0.36561 |
| *Elizabethkingia meningoseptica* | FCB group | Bacteroidetes/Chlorobi group | 4.01982 | 6 | 0.1982 | 4.8000E-03 | 0.69436 |
| *Enterobacter cloacae* | Proteobacteria | Gammaproteobacteria | 5.5988 | 25 | 0.1626 | 2.7194E-04 | 0.32504 |
| *Enterococcus faecalis* | Terrabacteria group | Firmicutes | 2.93304 | 25 | 0.1457 | 3.8187E-05 | 0.01406 |
| *Erwinia amylovora* | Proteobacteria | Gammaproteobacteria | 3.83383 | 13 | 0.1484 | 6.8000E-03 | 0.00660 |
| *Escherichia coli* | Proteobacteria | Gammaproteobacteria | 5.43741 | 25 | 0.1984 | 3.1165E-04 | 0.07089 |
| *Finegoldia magna* | Terrabacteria group | Firmicutes | 1.98674 | 6 | 0.1369 | 2.1236E-04 | 0.19218 |
| *Flavobacterium psychrophilum* | FCB group | Bacteroidetes/Chlorobi group | 2.86038 | 10 | 0.2493 | 1.3900E-02 | 0.00618 |
| *Fusobacterium nucleatum* | Fusobacteria | Fusobacteriia | 2.51403 | 12 | 0.2044 | 3.1494E-05 | 0.23476 |
| *Gallibacterium anatis* | Proteobacteria | Gammaproteobacteria | 2.69414 | 14 | 0.1413 | 4.6688E-05 | 0.09680 |
| *Gardnerella vaginalis* | Terrabacteria group | Actinobacteria | 1.73225 | 15 | 0.2060 | 3.8000E-03 | 0.61509 |
| *Haemophilus influenzae* | Proteobacteria | Gammaproteobacteria | 1.83014 | 7 | 0.0884 | 2.5738E-04 | 0.07925 |
| *Hafnia alvei* | Proteobacteria | Gammaproteobacteria | 4.71272 | 6 | 0.1458 | 1.4000E-03 | 0.48528 |
| *Helicobacter pylori* | Proteobacteria | delta/epsilon subdivisions | 1.66787 | 25 | 0.1345 | 1.3316E-05 | 0.16545 |
| *Lactobacillus paracasei* | Terrabacteria group | Firmicutes | 2.92433 | 14 | 0.1751 | 6.0845E-04 | 0.03190 |
| *Lactococcus lactis* | Terrabacteria group | Firmicutes | 2.36559 | 22 | 0.1943 | 9.9283E-05 | 0.13071 |
| *Legionella pneumophila* | Proteobacteria | Gammaproteobacteria | 3.39775 | 20 | 0.1304 | 1.1944E-04 | 0.06504 |
| *Leptospira interrogans* | Spirochaetes | Spirochaetia | 4.69813 | 25 | 0.1562 | 9.6629E-05 | 0.01934 |
| *Leuconostoc mesenteroides* | Terrabacteria group | Firmicutes | 2.07576 | 10 | 0.1905 | 3.6029E-04 | 0.02861 |
| *Listeria monocytogenes* | Terrabacteria group | Firmicutes | 2.94453 | 25 | 0.1173 | 3.4331E-04 | 0.16093 |
| *Mannheimia haemolytica* | Proteobacteria | Gammaproteobacteria | 2.73187 | 17 | 0.1101 | 4.6426E-04 | 0.13807 |
| *Melissococcus plutonius* | Terrabacteria group | Firmicutes | 2.06873 | 12 | 0.0596 | 4.3076E-04 | 0.00251 |
| *Metallosphaera sedula* | TACK group | Crenarchaeota | 2.19152 | 7 | 0.0024 | 1.88E-06 | 0.00001 |
| *Methanosarcina mazei* | Euryarchaeota | Methanomicrobia | 4.09635 | 15 | 0.0895 | 3.80E-05 | 0.02094 |
| *Micrococcus luteus* | Terrabacteria group | Actinobacteria | 2.5011 | 6 | 0.1390 | 2.7114E-04 | 0.05256 |
| *Microcystis aeruginosa* | Terrabacteria group | Cyanobacteria | 5.84279 | 8 | 0.2958 | 4.7194E-04 | 0.09152 |
| *Moraxella catarrhalis* | Proteobacteria | Gammaproteobacteria | 1.86329 | 12 | 0.1050 | 3.7396E-05 | 0.01658 |
| *Morganella morganii* | Proteobacteria | Gammaproteobacteria | 3.79954 | 8 | 0.1535 | 9.7104E-05 | 0.17277 |
| *Mycobacterium abscessus* | Terrabacteria group | Actinobacteria | 5.09049 | 25 | 0.1510 | 1.0000E-03 | 0.04481 |
| *Myroides odoratimimus* | FCB group | Bacteroidetes/Chlorobi group | 4.13866 | 7 | 0.0993 | 5.3817E-05 | 0.06442 |
| *Myxococcus xanthus* | Proteobacteria | delta/epsilon subdivisions | 9.13976 | 12 | 0.0293 | 2.1062E-06 | 0.00074 |
| *Neisseria meningitidis* | Proteobacteria | Betaproteobacteria | 2.27236 | 25 | 0.1225 | 8.2554E-04 | 0.07468 |
| *Neorhizobium galegae* | Proteobacteria | Alphaproteobacteria | 6.45503 | 9 | 0.1989 | 4.1299E-04 | 0.24319 |
| *Nonlabens ulvanivorans* | FCB group | Bacteroidetes/Chlorobi group | 3.21139 | 6 | 0.1607 | 3.0928E-05 | 0.26480 |
| *Oenococcus oeni* | Terrabacteria group | Firmicutes | 1.78052 | 13 | 0.1267 | 6.2224E-05 | 0.01273 |
| *Paenibacillus polymyxa* | Terrabacteria group | Firmicutes | 6.23851 | 10 | 0.1267 | 2.3198E-04 | 0.24409 |
| *Pantoea ananatis* | Proteobacteria | Gammaproteobacteria | 4.90814 | 12 | 0.1423 | 8.7372E-04 | 0.27359 |
| *Pasteurella multocida* | Proteobacteria | Gammaproteobacteria | 2.25749 | 21 | 0.1196 | 1.7000E-03 | 0.03375 |
| *Pectobacterium carotovorum* | Proteobacteria | Gammaproteobacteria | 4.86291 | 10 | 0.0867 | 1.5219E-04 | 0.15153 |
| *Pediococcus acidilactici* | Terrabacteria group | Firmicutes | 1.94283 | 6 | 0.1809 | 1.9000E-03 | 0.05111 |
| *Peptoclostridium difficile* | Terrabacteria group | Firmicutes | 4.29813 | 25 | 0.0010 | 4.9205E-05 | 0.02452 |
| *Photobacterium angustum* | Proteobacteria | Gammaproteobacteria | 5.18154 | 11 | 0.0994 | 2.1772E-04 | 0.05174 |
| *Pluralibacter gergoviae* | Proteobacteria | Gammaproteobacteria | 5.48968 | 10 | 0.1080 | 6.4252E-05 | 0.03616 |
| *Porphyromonas gingivalis* | FCB group | Bacteroidetes/Chlorobi group | 2.34348 | 18 | 0.1689 | 1.2029E-04 | 0.03017 |
| *Prochlorococcus marinus* | Terrabacteria group | Cyanobacteria | 1.74534 | 25 | 0.1983 | 3.0525E-04 | 1.17233 |
| *Propionibacterium acnes* | Terrabacteria group | Actinobacteria | 2.56026 | 25 | 0.0797 | 6.0107E-05 | 0.01871 |
| *Providencia alcalifaciens* | Proteobacteria | Gammaproteobacteria | 4.32682 | 9 | 0.1382 | 7.8576E-05 | 0.29821 |
| *Pseudomonas syringae* | Proteobacteria | Gammaproteobacteria | 6.0937 | 21 | 0.1852 | 6.6518E-04 | 0.41601 |
| *Ralstonia solanacearum* | Proteobacteria | Betaproteobacteria | 5.81092 | 9 | 0.1264 | 1.1339E-04 | 0.12948 |
| *Raoultella ornithinolytica* | Proteobacteria | Gammaproteobacteria | 6.09446 | 7 | 0.1323 | 5.3742E-04 | 0.22286 |
| *Rhizobium leguminosarum* | Proteobacteria | Alphaproteobacteria | 6.51806 | 9 | 0.4819 | 1.1000E-03 | 0.77911 |
| *Rhodobacter capsulatus* | Proteobacteria | Alphaproteobacteria | 3.87192 | 9 | 0.1000 | 2.2835E-04 | 0.09868 |
| *Rhodococcus erythropolis* | Terrabacteria group | Actinobacteria | 6.89554 | 7 | 0.1165 | 5.8663E-04 | 0.09221 |
| *Rhodopseudomonas palustris* | Proteobacteria | Alphaproteobacteria | 6.12396 | 11 | 0.4797 | 1.1000E-03 | 0.73413 |
| *Riemerella anatipestifer* | FCB group | Bacteroidetes/Chlorobi group | 2.30952 | 10 | 0.4137 | 9.1000E-03 | 0.07984 |
| *Ruminiclostridium thermocellum* | Terrabacteria group | Firmicutes | 3.8433 | 7 | 0.0781 | 3.5570E-04 | 0.00434 |
| *Salmonella enterica* | Proteobacteria | Gammaproteobacteria | 4.95137 | 25 | 0.1269 | 1.4132E-04 | 0.03419 |
| *Serratia marcescens* | Proteobacteria | Gammaproteobacteria | 4.85822 | 19 | 0.1453 | 2.8610E-04 | 0.17836 |
| *Shewanella baltica* | Proteobacteria | Gammaproteobacteria | 5.36877 | 10 | 0.1066 | 4.4210E-05 | 0.09518 |
| *Shigella flexneri* | Proteobacteria | Gammaproteobacteria | 4.82882 | 25 | 0.2021 | 1.6256E-04 | 0.01483 |
| *Sinorhizobium meliloti* | Proteobacteria | Alphaproteobacteria | 6.69169 | 7 | 0.0623 | 1.0883E-04 | 0.00552 |
| *Staphylococcus aureus* | Terrabacteria group | Firmicutes | 2.78256 | 25 | 0.0948 | 4.6420E-04 | 0.11744 |
| *Stenotrophomonas maltophilia* | Proteobacteria | Gammaproteobacteria | 3.50829 | 12 | 0.1567 | 2.4697E-04 | 0.26922 |
| *Streptococcus agalactiae* | Terrabacteria group | Firmicutes | 2.16027 | 25 | 0.1226 | 1.0519E-04 | 0.01406 |
| *Sulfolobus islandicus* | TACK group | Crenarchaeota | 2.73627 | 7 | 0.1153 | 1.52E-04 | 0.01605 |
| *Thermotoga maritima* | Thermotogae | Thermotogae | 1.86072 | 6 | 0.0083 | 4.7499E-05 | 0.00006 |
| *Ureaplasma urealyticum* | Terrabacteria group | Tenericutes | 0.94717 | 8 | 0.0726 | 1.6110E-04 | 0.01253 |
| *Vibrio parahaemolyticus* | Proteobacteria | Gammaproteobacteria | 5.16577 | 10 | 0.1403 | 6.12E-04 | 0.03439 |
| *Xanthomonas axonopodis* | Proteobacteria | Gammaproteobacteria | 5.29652 | 12 | 0.1436 | 4.2140E-04 | 0.09583 |
| *Xylella fastidiosa* | Proteobacteria | Gammaproteobacteria | 2.73301 | 13 | 0.1595 | 9.3476E-04 | 0.11352 |
| *Yersinia pestis* | Proteobacteria | Gammaproteobacteria | 4.82986 | 16 | 0.1108 | 3.1458E-05 | 0.00030 |
| *Zymomonas mobilis* | Proteobacteria | Alphaproteobacteria | 2.06141 | 8 | 0.0569 | 4.0920E-04 | 0.50053 |

* ffn files were kindly provided by Ben Temperton, ** ffn files from MV.

Table S2. *Pseudomonas* species

| *Pseudomonas* species | phi | var | pi (syn) |
| --- | --- | --- | --- |
| *Pseudomonas aeruginosa* | 0.0738 | 0.000013032 | 0.0229 |
| *Pseudomonas amygdali* | 0.1754 | 0.00036314 | 0.18015 |
| *Pseudomonas chlororaphis* | 0.3329 | 0.03670000 | 0.29699 |
| *Pseudomonas fluorescens* | 0.2611 | 0.000097912 | 0.62385 |
| *Pseudomonas mendocina* | 0.1647 | 0.00029257 | 0.39465 |
| *Pseudomonas psychrotolerans* | 0.0992 | 0.00130000 | 0.16837 |
| *Pseudomonas putida* | 0.228 | 0.00093062 | 0.45481 |
| *Pseudomonas savastanoi* | 0.1879 | 0.00086137 | 1.06905 |
| *Pseudomonas stutzeri* | 0.2251 | 0.00062268 | 0.65386 |
| *Pseudomonas syringae* | 0.1852 | 0.00066518 | 0.41601 |
| *Pseudomonas syringae* group genomosp. 3 | 0.1896 | 0.00088545 | 0.30621 |

Table S3. *Streptococcus* species

| *Streptococcus* species | phi | var | pi (syn) |
| --- | --- | --- | --- |
| *Streptococcus agalactiae* | 0.1226 | 0.00010519 | 0,01406 |
| *Streptococcus anginosus* | 0.165 | 0.0001704 | 0,17523 |
| *Streptococcus constellatus* | 0.1328 | 0.00082116 | 0,06936 |
| *Streptococcus dysgalactiae* | 0.1565 | 0.00026546 | 0,04444 |
| *Streptococcus equi* | 0.0476 | 0.00031707 | 0,01881 |
| *Streptococcus equinus* | 0.2061 | 0.00094923 | 0,57007 |
| *Streptococcus gallolyticus* | 0.187 | 0.00350000 | 0,07519 |
| *Streptococcus iniae* | 0.0641 | 0.00023344 | 0,00025 |
| *Streptococcus intermedius* | 0.1646 | 0.00067779 | 0,22561 |
| *Streptococcus mitis* | 0.213 | 0.000053146 | 0,41762 |
| *Streptococcus mutans* | 0.0994 | 0.000022041 | 0,02113 |
| *Streptococcus oralis* | 0.1814 | 0.00010873 | 0,26014 |
| *Streptococcus parasanguinis* | 0.1534 | 0.000043284 | 0,20533 |
| *Streptococcus pneumoniae* | 0.1703 | 0.000074265 | 0,0376 |
| *Streptococcus pseudopneumoniae* | 0.1154 | 0.000058014 | 0,10895 |
| *Streptococcus pyogenes* | 0.1438 | 0.001500000 | 0,02387 |
| *Streptococcus salivarius* | 0.1445 | 0.00010191 | 0,2012 |
| *Streptococcus sanguinis* | 0.1538 | 0.00023123 | 0,41192 |
| *Streptococcus suis* | 0.2074 | 0.00220000 | 0,09594 |
| *Streptococcus thermophilus* | 0.1147 | 0.00016554 | 0,01526 |
| *Streptococcus uberis* | 0.2074 | 0.00220000 | 0,23902 |

Table S4. Φ and r/m values for 25 species. r/m values were obtained from Vos and Didelot (2009)

| Species | phi | var | r/m |
| --- | --- | --- | --- |
| *Bacillus cereus* | 0.216 | 0.00005180 | 0.7 |
| *Bacillus thuringiensis* | 0.2264 | 0.000045851 | 0.8 |
| *Campylobacter jejuni* | 0.1336 | 0.00012610 | 2.2 |
| *Chlamydia trachomatis* | 0.0381 | 0.00013147 | 0.3 |
| *Enterococcus faecalis* | 0.1457 | 0.000038187 | 0.6 |
| *Escherichia coli* | 0.1984 | 0.00031165 | 0.7 |
| *Flavobacterium psychrophilum* | 0.2493 | 0.01390000 | 63.6 |
| *Haemophilus influenzae* | 0.0884 | 0.00025738 | 3.7 |
| *Helicobacter pylori* | 0.1345 | 0.000013316 | 13.6 |
| *Legionella pneumophila* | 0.1304 | 0.00011944 | 0.9 |
| *Leptospira interrogans* | 0.1562 | 0.000096629 | 0.1 |
| *Listeria monocytogenes* | 0.1173 | 0.000343310 | 0.7 |
| *Microcystis aeruginosa* | 0.2958 | 0.000471940 | 18.3 |
| *Moraxella catarrhalis* | 0.105 | 0.000037396 | 10.1 |
| *Myxococcus xanthus* | 0.0293 | 0.000002106 | 5.5 |
| *Neisseria meningitidis* | 0.1225 | 0.000825540 | 7.1 |
| *Oenococcus oeni* | 0.1267 | 0.000062224 | 0.7 |
| *Porphyromonas gingivalis* | 0.1689 | 0.00012029 | 0.4 |
| *Pseudomonas syringae* | 0.1852 | 0.00066518 | 1.5 |
| *Ralstonia solanacearum* | 0.1264 | 0.00011339 | 1.1 |
| *Salmonella enterica* | 0.1269 | 0.00014132 | 30.2 |
| *Staphylococcus aureus* | 0.0948 | 0.00046420 | 0.1 |
| *Streptococcus pneumoniae* | 0.1703 | 0.00007426 | 23.1 |
| *Streptococcus pyogenes* | 0.1438 | 0.00150000 | 17.2 |
| *Sulfolobus islandicus* | 0.1153 | 0.00015195 | 1.2 |
| *Vibrio* *parahaemolyticus* | 0.1403 | 0.000612160 | 39.8 |

**Supplemental Figure 1**

The genome fluidity φ statistic calculated for both closed- and high-quality (<150 contigs) draft genomes for 31 species based on sets of 9-25 genomes.

**
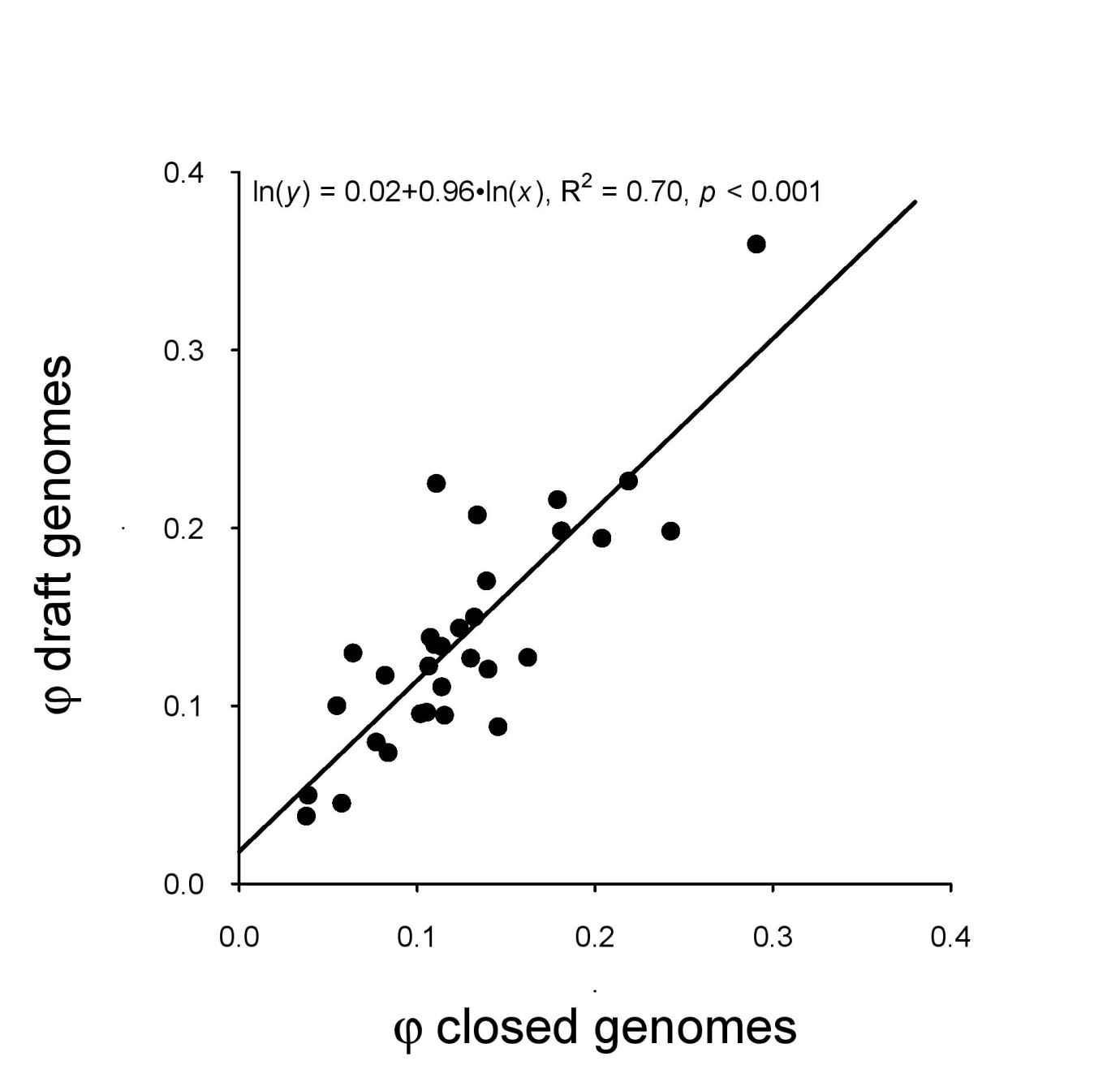
**

**Supplemental Figure 2**

The relationship between synonymous core genome diversity (π_syn_, ln scale) and accessory genome fluidity (φ, ln scale) plotted for the complete data set (minus the single *Pseudomonas* and *Streptococcus* species used in Figure 1) (filled dots) and the genera *Pseudomonas* (diamonds) and *Streptococcus* (open dots). The line and shaded area depict the fitted relationship and 95% prediction interval based on the complete data set, where (linear regression: ln(ϕ)=-1.35+0.26*ln(π_syn_), a: *t=*-13.68*** and b: *t*=9.47***, adjusted R^2^=0.49).

**
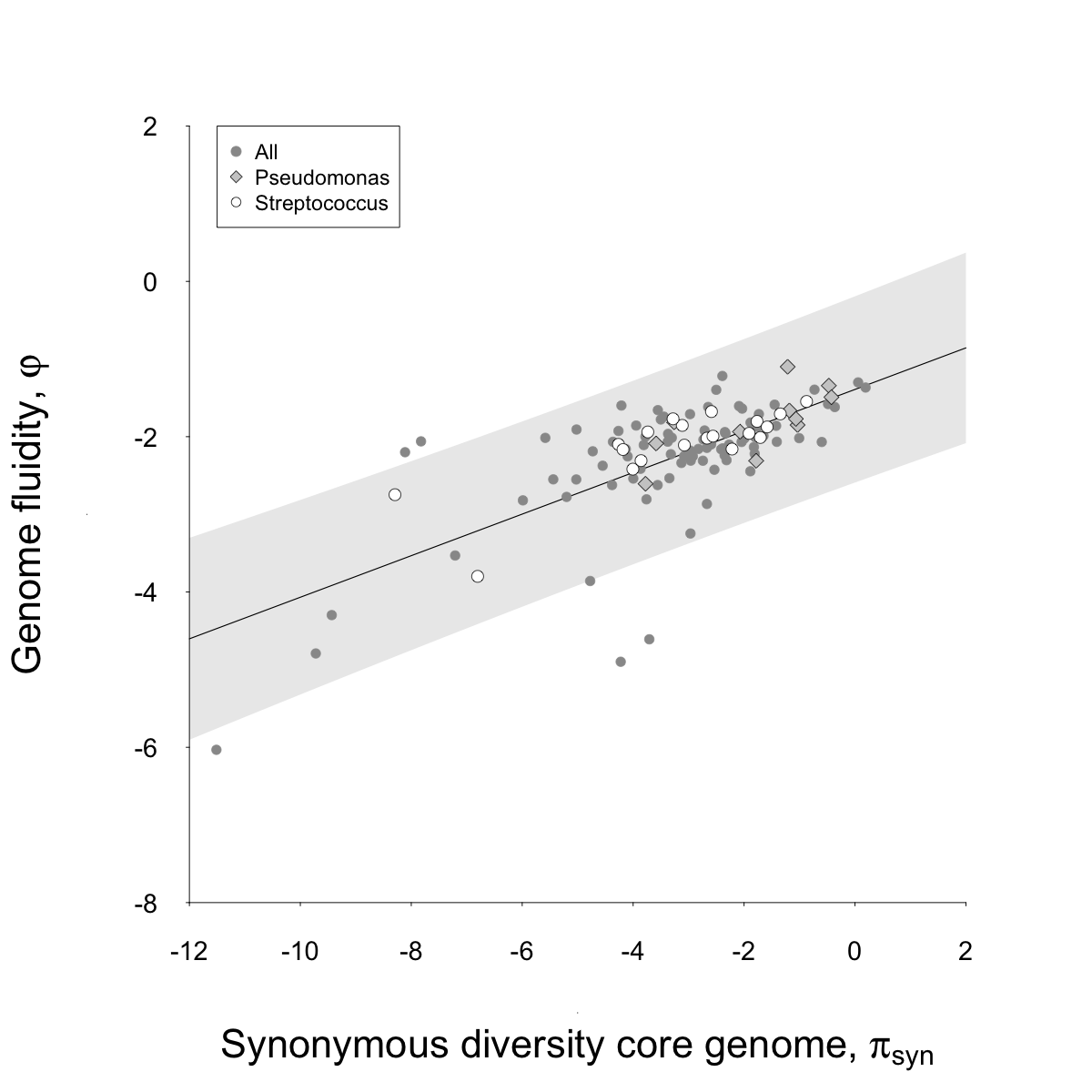
**

**Supplemental References**

Abascal F, Zardoya R, Telford MJ (2010). TranslatorX: multiple alignment of nucleotide sequences guided by amino acid translations. *Nucl Acids Res* 38: W7-W13.

Edgar RC (2004). MUSCLE: multiple sequence alignment with high accuracy and high throughput. *Nucl Acids Res* 32: 1792-1797.

Kumar S, Stecher G, Peterson D, Tamura K (2012). MEGA-CC: computing core of molecular evolutionary genetics analysis program for automated and iterative data analysis. *Bioinformatics* 28: 2685-2686.

Li L, Stoeckert CJ, Roos DS (2003). OrthoMCL: identification of ortholog groups for eukaryotic genomes. *Genome Res* 13: 2178-2189.

Team D (2008). Development Core Team. R: A language and environment for statistical computing. R Foundation for Statistical Computing, Vienna, Austria. ISBN 3-900051-07-0, URL http://www. R-project. org.

Vos M, te Beek TA, van Driel MA, Huynen MA, Eyre-Walker A, van Passel MW (2013). ODoSE: A Webserver for Genome-Wide Calculation of Adaptive Divergence in Prokaryotes. *Plos One* 8: e62447.
